# Sensitivity pattern among bacterial isolates in neonatal septicaemia in port Harcourt

**DOI:** 10.1186/1476-0711-11-7

**Published:** 2012-03-26

**Authors:** Boma A West, Oliemen Peterside

**Affiliations:** 1 Department of Paediatrics and Child Health, Braithewaite Memorial Specialist Hospital, Port Harcourt, Rivers State, Nigeria; 2 Department of Paediatrics and Child Health, Niger Delta University Teaching Hospital, Okolobiri, Bayelsa State, Nigeria

**Keywords:** Neonatal sepsis, Bacteria, Antibiotics, Port Harcourt

## Abstract

**Background:**

The organisms responsible for neonatal sepsis vary across geographical boundaries and with the time of illness thus periodic bacteriologic surveillance is a neccessity. The present study was therefore carried out to determine the common bacterial pathogens in Port Harcourt and their sensitivity pattern.

**Methods:**

Four hundred and six neonates were prospectively screened for sepsis over a 6 month period. Sensitivity of the bacterial isolates to different antibiotics was determined using Kirby-Bauer diffusion method.

**Results:**

Gram negative organisms predominated (75.1%) with *Klebsiella pneumonia *(58.2%) being the commonest. The quinolones were the most sensitive antibiotics to the commonly isolated organisms.

**Conclusion:**

*Klebsiella pneumonia *is the commonest organism responsible for neonatal sepsis in Port Harcourt. There is an overall decline in the antibiotic susceptibility to the commonly isolated bacterial pathogens.

## Introduction

Sepsis remains one of the most common diseases of the neonatal period and is still a significant cause of morbidity and mortality [1]. It contributes up to 13-15% of all deaths during the neonatal period, higher in developing countries where it contributes between 30-50% [2,3]. It is important to note that 20-30% of the survivors of neonatal sepsis may exhibit neurological sequalae [4]. Sepsis related mortality is however largely preventable with rational antimicrobial therapy and aggressive supportive care [5].

The organisms responsible for neonatal sepsis (NNS) vary across geographical boundaries and with the time of onset of illness [6]. In addition, one organism or a group of organisms may over time replace another as the leading cause of neonatal sepsis in a particular region [1,7,8]. In most developing countries, gram negative bacteria remain the major source of infection [9]. However, in the developed countries, Gram positive organisms have been implicated as the most common causes of NNS [10].

Micro-organisms implicated in NNS have developed increased drug resistance to commonly used antibiotics and thus making treatment extremely difficult [11]. Thus, local epidemiology of neonatal sepsis should be constantly updated to detect changes in the pattern of infection of pathogens and their susceptibility to various antibiotics. The epidemiology of NNS and antibiotic resistance patterns of pathogens may be used to develop guidelines for management of NNS in hospitals including the choice of empiric antibiotic therapy. The aim of this study therefore is to determine the most common bacterial pathogens associated with the disease and their antimicrobial susceptibility.

## Methods

The study was carried out prospectively over a 6 months period (July-December, 2007) in the Special Care Baby Unit of the University of Port Harcourt Teaching Hospital, Rivers State, Nigeria. The University of Port Harcourt Teaching Hospital is the main referral and neonatal care centre for Rivers State and its neighboring States.

All newborns aged 0-28 days admitted during the period of study with one or more symptoms/signs suggestive of sepsis with or without risk factors of sepsis were recruited into the study. Babies who had received antibiotics prior to presentation as well as those whose mothers had received antibiotics within one week prior to delivery were excluded from the study. For every neonate recruited, 2 milliliter of venous blood was collected from a peripheral vein under aseptic conditions and before the commencement of antibiotics for blood culture. The blood was aseptically introduced into aerobic and anaerobic culture media. The blood culture specimens were processed according to standard methods in the microbiology laboratory [12]. Inoculated blood culture media were considered negative if there was no growth after continuous incubation for up to 7 days, subcultures being made each day. Sensitivity of the bacterial isolates to different antibiotics was determined using Kirby-Bauer disc diffusion method [12].

Neonates whose samples for investigations had been sent to the laboratories were commenced empirically on intravenous cloxacillin and gentamicin, based on previous antibiotic sensitivity pattern. Clinical response was monitored and therapy changed to another antibiotic (cephalosporins) if response was poor or patient was deteriorating. Clinical response was said to be poor when there was no improvement in the symptoms and signs after 72 hours of antibiotic treatment. In the case of a positive blood culture, the 2nd line antibiotic chosen was determined by the susceptibility pattern of the organism isolated.

The clinical details and results of laboratory investigations were recorded in a proforma. The results were analysed using the statistical package, SPSS version 14.0. Ethical clearance was obtained from the ethics committee of the University of Port-Harcourt Teaching Hospital.

## Results

Five hundred and eleven neonates were admitted into the SCBU of the UPTH during the period of study, of which 406 (79.5%) who had clinical features suggestive of sepsis with or without risk factors of sepsis were studied. Of the 406 neonates, 153 (37.7%) were inborns while 253 (62.3%) were outborns.

Of the 406 neonates studied 169 (41.6%) had positive blood culture, giving a prevalence rate of blood culture proven sepsis as 33.1%.

### Organisms Isolated from blood culture

**Figure 1:**
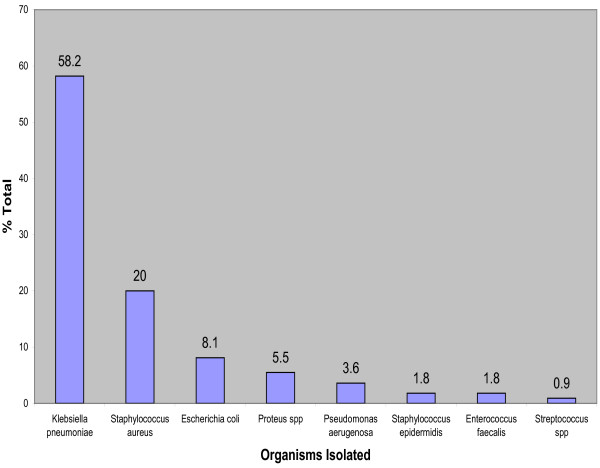
**Organisms Isolated from Blood Culture**.

As illustrated in Figure 1, there was a preponderance of gram negative organisms, 127 (75.1%) over gram positive organisms, 42 (24.9%). *Kebsiella pneumoniae *(58.2%) was the commonest bacterial pathogen isolated followed by *Staphylococcus aureus *(20.0%) and *Escherichia coli *(8.1%) while the least organism isolated was Streptococcus spp (0.9%).

### Organisms isolated by age at onset of illness

The distribution of the organisms isolated from blood culture by age at onset of illness is illustrated in Table 1. There were 120 (42.1%) neonates with Early onset sepsis, EOS (onset of illness in the first 72 hours of life) and 49 (40.5%) with Late onset sepsis, LOS (onset of illness after 72 hours of life), p value = 0.76. *Klebsiella pneumonia *(65.4%) *and Staphylococcus aureus *(15.4%) were the commonest organisms isolated in neonates with EOS while the least were *Pseudomonas aerugenosa *(2.6%) and *Enterococcus faecalis *(2.6%). *Staphylococcus epidermidis *(0.0%) and Streptococcus spp (0.0%) were not isolated in neonates with EOS.

**Table 1:** Organisms isolated by age at onset of illness

Organisms	Early onset sepsis (0-72 hrs)	Late onset sepsis (> 72 hrs)
	**Frequency [Percentage]**	**Frequency [Percentage]**

Klebsiella	78 (65.0)	21 (42.9)
Staphylococcus aureus	18 (15.0)	15 (30.6)
Escherichia coli	9 (7.5)	4 (8.2)
Proteus spp	8 (6.7)	1 (2.0)
Pseudomonas aurugenosa	4 (3.3)	4 (8.2)
Enterococcus faecalis	3 (2.5)	0 (0)
Staphylococcus epidermidis	0 (0)	3 (6.1)
Streptococcus spp	0 (0)	1 (2.0)
Total	120 (100)	49 (100)

For late onset sepsis, *Klebsiella pneumonia *(43.6%) and *Staphylococcus aureus *(30.8%) were also the predominant organisms implicated while the least were Proteus spp (2.6%) and Streptococcus spp (2.6%). *Enterococcus faecalis *(0.0%) was not isolated in neonates with LOS.

### Isolation of organisms by gestational age at birth

**Figure 2:**
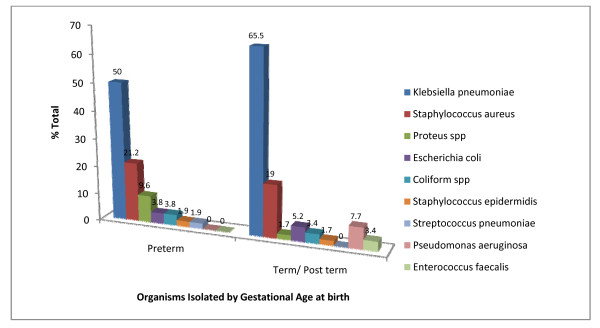
**Organisms Isolated by Gestational Age at Birth**.

Figure 2 illustrates the distribution of organisms isolated by gestational age. *Klebsiella pneumonia *(50.0%) and *Staphylococcus aureus *(21.2%) were the predominant organisms isolated in preterm neonates while *Staphylococcus epidermidis *(1.9%) and Pseudomonas spp (1.9%) were the least isolated.

In Term/Post term neonates, *Klebsiella pneumonia *(65.5%) and *Staphylococcus aureus *(19.0%) were also isolated predominantly, while Proteus spp (1.7%) and *Staphylococcus epidermidis *(1.7%) were the least isolated.

### Antibiotic susceptibility testing of *Klebsiella pneumoniae*

**Figure 3:**
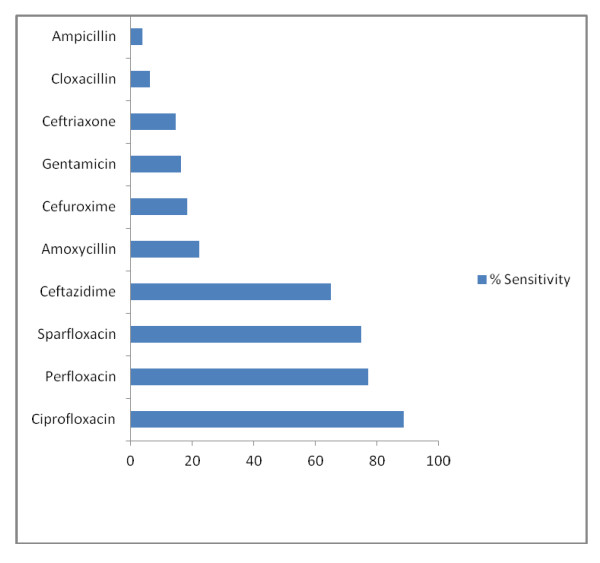
**Graphical Representation of *Klebsiella pneumoniae *Susceptibility Testing**.

The graphical representation of *Klebsiella pneumoniae *susceptibility testing is illustrated in Figure 3. Ciprofloxacin (88.5%), perfloxacin (77.1%) and sparfloxacin (77.1%) were the most sensitive antibiotics to *Klebsiella pneumoniae *while the least were cloxacillin (6.3%) and ampicillin (3.8%).

### Antibiotic sensitivity testing of *Staphylococcus aureus*

**Figure 4:**
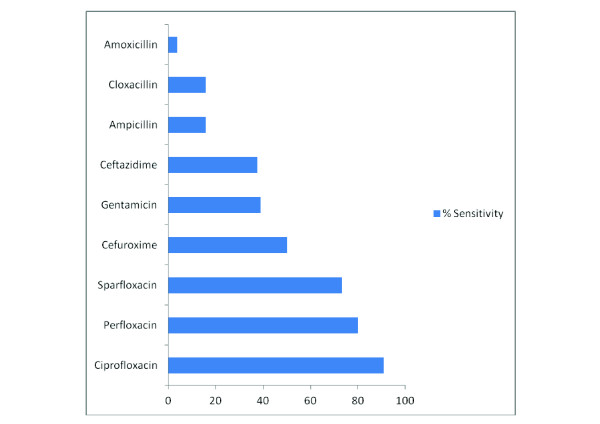
**Graphical Representation of *Staphylococcal aureus *susceptibility pattern**.

The antibiotic sensitivity of *Staphylococcus aureus *is shown in Figure 4. *Staphylococcus aureus *was most sensitive to ciprofloxacin (90.9%) followed by perfloxacin (80.0%) and sparfloxacin (73.3%) but less sensitive to ampicillin (15.8%), cloxacillin (15.8%) and amoxicillin (3.8%).

## Discussion

Sepsis remains one of the most important causes of morbidity and mortality in the newborn despite considerable progress in hygiene, introduction of new antimicrobial agents and advanced measures for early diagnosis and treatment [13,14].

The burden of neonatal sepsis in our environment is enormous as shown in the present study in which 79.5% of all neonates admitted had either features suggestive of sepsis or predisposing factors of sepsis and a third of this number had blood culture proven sepsis. Similar observation was also made in Jos, Nigeria [15].

The high prevalence of sepsis of 33.1% in the present study corroborates with the 31.7% and 34.4% reported in Calabar and Jos, Nigeria [2,15] respectively but much higher than the 10.7% and 6.5% reported in ogun state, Nigeria [16] and London [10] respectively. The higher prevalence observed in the present study could be attributed to the fact that the study was prospective and as such infants of mothers with prior antibiotic therapy were excluded from the study. This may not have been the case in the latter studies which were retrospective. The lower prevalence rate observed in London [17] could also be attributed to the better obstetric and nursery care as seen in other developed countries [8].

Neonatal sepsis is a life threatening emergency and thus any delay in treatment may cause death. The knowledge of the aetiological organisms as well as their antimicrobial sensitivity profile is necessary for effective therapeutic intervention in neonatal sepsis. It is therefore important to note that commencement of antibiotic therapy empirically is of essence while awaiting blood culture results. The initial empiric antibiotic therapy must therefore be a combination of drugs to cover for the prevalent bacterial organisms in that locality.

In the present study, Gram negative organisms predominated accounting for 75.1% of neonatal septicaemia in our unit. Similar finding has been reported in most parts of Nigeria [16,18-20] and India [21]. This however contrasts with studies carried out in the non-tropics where Gram positive organisms predominated [10].

The predominance of *Klebsiella pneumonia *in the present study accords with several reports in Nigeria [16,18,20] and other developing countries [17,22]. This however contrast with studies in some parts of Nigeria [7,23,24] and Saudi Arabia [25] where *Staphylococcus aureus *was mainly implicated. These differences could be attributed to geographic location and with the time of onset of illness. In addition, one organism or a group of organisms may over time replace another as the leading cause of neonatal sepsis in a particular region [7,8,10].

*Klebsiella pneumonia *was observed in the present study to be the commonest organism implicated in neonates with both EOS and LOS. Not surprisingly however, *Staphylococcus aureus *was noted to be more implicated in LOS than in EOS. This organism was also observed more in the preterms than in the term/post term neonates with sepsis. This could be because *Staphylococcus aureus *is commonly associated with nosocomial sepsis as seen in LOS as well as in immunocompromised patients like the preterm babies.

It is interesting to note that isolation of *Group B Streptococcus *was very insignificant in the present study and this confirms the observation by researchers in Nigeria [2,3,15,18,19] and other developing countries [17,21,22]. The low incidence of GBS sepsis in developing countries could be attributable to low prevalence of GBS colonization rates of pregnant women or possibly to the presence of strains with low virulence

The present study has shown a change in the sensitivity pattern of the common pathogens to commonly used antibiotics. Quinolones (ciprofloxacin, perfloxacin and sparfloxacin) were observed to be the most potent antimicrobial agents against both Gram negative and positive organisms in our unit and least sensitive to ampicillin and cloxacillin commonly used in the SCBU of our hospital. This corroborates with other studies [18,21,24,26]. The 3rd generation cephalosporins now commonly used as 2nd line antibiotics in many centres and 1st line in others have recently also been observed to become increasingly ineffective as shown in the present study as well as others [18,26]. The sensitivity to gentamicin in the present study was however poor, 16.3% for *Klebsiella pneumonia *and 38.9% for *Staphylococcus aureus*. Progressive decline in the sensitivity pattern of gentamicin to gram negative organisms has also been observed by other researchers [3,7,16,20]. This however contrast much earlier studies where gentamicin was observed to be very sensitive to commonly isolated organisms [19,27]. This change in the sensitivity pattern of antimicrobials could be attributable to the fact that microorganisms tend to become resistant to commonly used antibiotics while remaining sensitive to the rarely used ones. In addition, antimicrobial sensitivity may differ in studies and at different times and this could be due to the emergence of resistant strains due to indiscriminate use of antibiotics for both prophylaxis and treatment of sick neonates.

Based on the present study, it is probably unnecessary to commence cloxacillin and gentamicin previously recommended as empiric antibiotics in the treatment of neonates with suspected sepsis due to their extremely low sensitivities. Evidence from the present study has shown that the quinolones especially ciprofloxacin is the most potent antibiotics in neonatal sepsis. These however have restricted use in children although there has been successful short term use of the drug in cases of severe NNS [28]. Because of the dreaded side effects of the quinolones, the 3rd generation cephalosporins which had moderate sensitivities may be advocated as first line antibiotics in the treatment of neonates with sepsis. The major limiting factor of the cephalosporins however, is its prohibitive cost. In the face of gentamicin resistance, other aminoglycosides not commonly used like amikacin and tobramycin may be recommended as alternatives. It is thus pertinent to note that the current antibiotic policy of commencing a baby with suspected sepsis on cloxacillin and gentamicin needs re-evaluation.

The varying microbiological pattern of NNS therefore warrants the need for periodic review of neonatal sepsis as the knowledge of the pathogens and their antibiotic susceptibility would be a useful guide in the antibiotic therapy of such neonates with sepsis.

Futhermore, steps need be taken to prevent or control the emergence of resistance strains. Laws therefore should be enforced to discourage the indiscriminate use of antibiotics seen commonly in our country as well as discourage inadequate doses which are also believed to contribute to the increasing emergence of resistant strains.

## Conclusion

*Klebsiella pneumoniae *is the commonest organism implicated in neonates with sepsis.

The quinolones are the most potent antibiotics in neonatal sepsis.

## Competing interests

The authors declare that they have no competing interests.

## Authors' contributions

BAW and OP both carried out the study together. BAW wrote the initial manuscript which was reviewed by OP. Both authors read and approved the final manuscript.

## References

[B1] Chako B Sohi I Early Onset Neonatal Septicaemia Indian J Pediatr 2005 72 23 26 10.1007/BF02760574 15684443

[B2] Antia-Obong CE Utsalo SJ Udo JJ Udo KT Neonatal Septicaemia in Calabar, Nigeria Central Afr J Med 1992 36 161 165 1394397

[B3] Omene JA Neonatal Septicaemia in Benin city, Nigeria: a review of 74 cases Trop Geogr Med 1979 31 35 39 384626

[B4] Klein JO Marcy MS Remington JJ, Klein JO Bacterial Sepsis and Meningitis Infectious Diseases of the Fetus and the Newborn 1995 Philadelphia: WB Saunders Co 835 890

[B5] Sanker MJ Agarwal R Deorari AK Paul VK Sepsis in the Newborn Indian J Pediatr 2008 75 261 266 10.1007/s12098-008-0056-z 18376095

[B6] Al-Zwaini EJK Neonatal Septicaemia in the Neonatal Care Unit, Al-Anbar governorate, Iraq East Medit Health J 2002 8 4 5 15603032

[B7] Amiebenomo CS Yakubu AM Bello CSS Ewa B Neonatal Septicaemia in Zaria Nig Med J 1988 18 349 351

[B8] Edwards MS Fanaroff AA, Martin RJ Postnatal Bacterial Infections Neonatal Perinatal Medicine: Diseases of the fetus and infant 2002 7 St Louis: CV Mosby 706 726

[B9] Klein JO Marcy MS Remington JS, Klein JO Bacterial Sepsis and Meningitis Infectious Diseases of the Fetus and the Newborn Infant 2001 5 Philadelphia: WB Saunders Co 943 998

[B10] Plazek MM Whitelaw A Early and Late Neonatal Septicaemia Arch Dis Child 1983 58 728 731 10.1136/adc.58.9.728 6625634 PMC1628213

[B11] Motara F Ballot DE Perovic O Epidemiology of Neonatal Sepsis at Johannesburg Hospital Southern Afr J Epidemiol Infect 2005 20 90 93

[B12] Antia-Obong OE Utsalo SJ Bacterial Agents in Neonatal Septicaemia in Calabar, Nigeria (A review of 100 cases) West Afr J Med 1993 12 114 117 8398930

[B13] Gotoff SP Behrman RE, Kleigman RM, Arvin AM Neonatal sepsis and meningitis Nelson Textbook of Pediatrics 1996 15 Philadelphia: WB Saunders Co 528 537

[B14] Haque KH Campbell AGM, Macintosh N Infection and Immunity in the newborn Forfor and Arneil's Textbook of pediatrics 1998 5 Pearson Professional Limited 273 289

[B15] Bode-Thomas F Ikeh EI Ejeliogu EU Current aetiology of neonatal sepsis in Jos University Teaching Hospital Nig J Med 2004 13 130 135 15293830

[B16] Njokanma CF Olanrewaju DM Akesode FA Antibiotic resistance among bacterial isolates in neonatal septicaemia Nig J Paediatr 1994 21 47 51

[B17] Zeeshan A Tariq G Talal W Salman A Shahid A Shahid M Diagnostic value of c-reactive protein and haematologic parameters in neonatal sepsis J Coll Physicians Surg Pak 2005 15 152 156 15808093

[B18] Iroha EO Egri-Okwaji MTC Kesah CN Odugbemi TO Changing pattern of causative organisms of neonatal septicaemia in Lagos University Teaching Hospital Nig J Paediatr 1998 25 1 5

[B19] Airede AI Neonatal septicaemia in an African City of high altitude J Trop Pediatr 1992 38 189 191 1527816 10.1093/tropej/38.4.189

[B20] Dawodu AH Alausa OK Neonatal septicaemia in the tropics Afr J Med Sci 1980 2 1 6 6282080

[B21] Roy I Jain A Kumar M Agarwal SK Bacteriology of Neonatal Septicaemia in a Tertiary Hospital of Northern India Indian J Med Microbiol 2002 20 156 159 17657057

[B22] Manucha V Rusia U Sikka M Faridi MMA Madan N Utility of haematological parameters and c-reactive protein in the detection of neonatal sepsis J Paediatr Child Health 2002 38 459 464 10.1046/j.1440-1754.2002.00018.x 12354261

[B23] Ugochukwu EF Bacterial isolates in neonatal infections Nig Med Pract 2003 44 56 58

[B24] Awoniyi DO Udo SJ Oguntibeju OO An epidemiological survey of neonatal sepsis in a hospital in Western Nigeria Afr J Microbiol Research 2009 3 385 389

[B25] Nuntnarumit P Pinkaew O Kitiwanwanich S Predictive values of serial c-reactive protein in neonatal sepsis J Med Assoc Thai 2002 85 1151 1158 12549789

[B26] Ebelechukwu FU Bacterial isolates in neonatal infections Nig Med Pract 2003 44 56 58

[B27] Dawodu A Twum-Danso K Al Umran K A case control study of neonatal sepsis: experience from Saudi Arabia J Trop Pediatr 1997 43 84 88 10.1093/tropej/43.2.84 9143177

[B28] Omokhodion SI The use of ofloxacin in infants with rapidly deteriorating Septicaemia and multiple antibiotic resistance Nig J Paediatr 1994 21 83 84

